# Supplementing semen extenders with a combination of phosphorus and vitamin B12 Improves post-thawed cryopreserved rooster semen quality

**DOI:** 10.3389/fvets.2023.1301186

**Published:** 2023-12-20

**Authors:** Junpen Suwimonteerabutr, Punnapon Ketkaew, Gitsanai Netiprasert, Chidchanok Khaopong, Boonyaporn Osiriphan, Pawarisa Sriamornrat, Morakot Nuntapaitoon

**Affiliations:** ^1^Department of Obstetrics, Gynecology and Reproduction, Faculty of Veterinary Science, Chulalongkorn University, Bangkok, Thailand; ^2^Faculty of Veterinary Science, Chulalongkorn University, Bangkok, Thailand; ^3^Multi-Omics for Functional Products in Food, Cosmetics and Animals, Research Unit, Bangkok, Thailand

**Keywords:** extender, frozen semen, phosphorus, Thai native rooster, vitamin B12

## Abstract

Semen cryopreservation is an important technique for preserving the genetic material of numerous species. However, frozen semen is highly susceptible to sperm DNA damage and reduced motility, resulting in decreased fertility. The standard method for cryopreservation and several approaches have not been elucidated. This study aimed to determine the effects of supplementing rooster semen extender with a combination of phosphorus and vitamin B12 on cryopreserved semen quality. Semen was collected weekly via dorso-abdominal massage from 57 Burmese × Vietnam-crossbred Thai native roosters aged 1–3 years. In total, 139 semen samples were collected, pooled, and diluted to 200 million sperm per dose. The pooled sample was divided into six experimental groups: a control group (0.00%) diluted with modified Beltville Poultry Semen Extender (BPSE) and five treatment groups diluted with modified BPSE supplemented with phosphorus and vitamin B12 at concentrations 0.02, 0.04, 0.06, 0.08, and 0.10%, respectively. The semen samples were frozen and evaluated at 0, 15, and 30 min after thawing. Sperm kinematic parameters were determined using a computer-assisted sperm analysis system. Sperm quality was evaluated by measuring sperm viability, mitochondrial activity, acrosome integrity, and plasma membrane integrity. Statistical analyses were performed using a general linear mixed model (MIXED) in SAS. Factors in the statistical model were experimental groups, time after thawing, and interaction between experimental groups and time after thawing. Total and progressive motilities were greater in semen supplemented with 0.04% phosphorus and vitamin B12 compared with those in the control (*p* < 0.05). At 15 min post-thawing, VCL, VAP, and HPA in the 0.04% phosphorus and vitamin B12 supplementation group was greater than that in the control (*p* < 0.05). Phosphorus and vitamin B12 supplementation did not affect sperm kinematics at 0 and 30 min after thawing (*p* > 0.05). All the sperm parameters that were tested for the 0.04% phosphorus and vitamin B12 supplementation group in modified BPSE were the highest at all the timepoints after thawing. Thus, supplementing frozen semen extender with 0.04% phosphorus and vitamin B12 increased sperm motility, sperm kinematic parameters, and sperm quality.

## 1 Introduction

Artificial insemination has been widely applied in poultry for overcoming low fertility, preventing infection transmission, and improving genetics (1, 2). Semen cryopreservation is an important technique for preserving the genetic material of numerous species (3–6). Cryopreserved semen can be used to considerably enhance genetic diversity, especially in animal populations with ongoing or that are at risk for inbreeding (5, 6). However, freeze-thaw cycles during the recovery of cryopreserved rooster semen can reduce sperm viability by compromising sperm cell membrane permeability and damaging the sperm mitochondria, midpiece, and acrosome (7). Particularly, avian spermatozoa have few cytoplasmic antioxidants and abundant polyunsaturated fatty acids in the membrane, rendering avian sperm extremely sensitive to oxidative stress during cryopreservation (8–11). Thus, frozen semen is highly susceptible to sperm DNA damage and reduced motility, resulting in decreased fertility (11, 12). However, to the best of our knowledge, there is no standard method for cryopreservation and several approaches have been reported in previous studies (13–19). However, this technique requires further refinement to increase success in its applications.

Phosphorus is a main energy source for sperm, with cyclic adenosine monophosphate (cAMP) serving as an important factor for sperm motility (20). In hamster sperm, cAMP-dependent phosphorylation of 36- and 65 kDa proteins plays a role in regulating the speed of microtubule motion (21). Furthermore, cyanocobalamin or vitamin B12 is a water-soluble vitamin that acts as a cofactor in numerous important biochemical pathways, such as methionine synthesis and branched-chain amino acid metabolism (22). Previous studies reported that vitamin B12 deficiency increases incomplete sperm formation and impairs sperm motility and velocity in rats (23). Supplementing chilled boar semen with phosphorus and vitamin B12 improved the total motility, progressive motility, sperm viability, and plasma membrane integrity of the sperm (24). In cattle and rams, supplementing semen extender with vitamin B12 improves the quality of thawed spermatozoa (25).

To the best of our knowledge, it is currently unknown whether phosphorus and vitamin B12 supplementation during cryopreservation can improve rooster sperm quality. Therefore, this study aimed to determine the effect of combined phosphorus and vitamin B12 supplementation in semen extender on the quality of frozen-thawed semen in Thai native rooster.

## 2 Methodology

The study protocol was approved by the Institutional Animal Care and Use Committee of the Faculty of Veterinary Science, Chulalongkorn University (Approval number 2131018) and complies with provisions of “The Ethical Principles and Guidelines for the Use of Animals for Scientific Purposes” (edited by the National Research Council of Thailand).

### 2.1 Animals

This study employed a case-control design which included 57 roosters in a local Thai native chicken farm in Thailand. In total, 139 ejaculates of semen were collected from Burmese × Vietnam-crossbred Thai native roosters aged between 1 and 3 years. Thai native roosters were kept in individual pens and fed *ad libitum* with paddy rice mixed with water.

### 2.2 Semen collection and experimental design

Semen was collected using the dorso-abdominal massage method weekly. The macroscopic and microscopic examinations of the rooster semen were performed. Semen samples with total motility of <65% were discarded. Following collection, semen was pooled and diluted to 200 million sperm per dose. The components of modified BPSE (26) were sodium glutamate (8.67 g/L), sodium acetate (0.43 g/L), magnesium chloride (0.34 g/L), potassium citrate (0.64 g/L), dipotassium phosphate (12.7 g/L), monopotassium phosphate (0.65 g/L), TES [*n*-tris (hydroxymethyl) methyl 1–2 amino ethane sulfonic acid] (1.95 g/L), Trehalose (1.9 g/L), Fructose (5 g/L) with pH of 7.5 and osmolarity of 366 mOsm/kg. The pooled semen was divided into six groups. The control group was diluted with modified Beltville Poultry Semen Extender (BPSE) plus 0.5% dimethyl sulfoxide (DMSO). The five treatment groups were diluted with modified BPSE plus 0.5% dimethyl sulfoxide (DMSO) and supplemented with various concentrations of phosphorus and vitamin B12 (Octafos^®^ Octa Memorial Co., Ltd., Bangkok, Thailand). Each pooled ejaculate (12 repetitions) was split into six equal aliquots. Then, put the completed rooster semen extenders [0.00% (Control), 0.02, 0.04, 0.06, 0.08, 0.10%] in randomly. The semen samples in microtubes were wrapped with tissue paper for slowly cooled and kept at 4°C during transportation (27).

### 2.3 Chemical

The supplementation solution was prepared by adding 100 mg butaphosphan, 0.05 cyanocobalamin mg, and 1 mg methyl paraben to 1 mL solution and diluted to achieve final concentrations of 0.02, 0.04, 0.06, 0.08, and 0.10%.

### 2.4 Cryopreservation and thawing

Chemical reagents were purchased from Sigma (St. Louis, MO, United States). The freezing procedure was performed as reported previously by Amini et al. (10) with some modifications. After arriving at the laboratory, the diluted semen samples underwent equilibration at 4°C for 2 h, followed by the addition of 3% glycerol and 0.5% dimethyl formamide. The samples were loaded into 0.25 mL. French straw (IMV, L’Aigle, France) incubated for 15 min in a cooled tray. The straws were placed at ~5 cm above the liquid nitrogen vapour for 15 min in a 40 × 20 × 20 cm styrofoam box containing 8,000 cm^3^ liquid nitrogen (28). After 1 week, the frozen straws were thawed for 3 min in a water bath at 5°C. Thawed samples were placed at room temperature (20–25°C). At 0, 15, and 30 min, samples were analyzed for sperm evaluation.

### 2.5 Sperm evaluation

#### 2.5.1 Computer-assisted sperm analysis

The semen samples were evaluated at 0, 15, and 30 min using the computer-assisted sperm analysis (CASA) system (SCA^®^, Microptic, Barcelona, Spain). The settings were adjusted to detecting avian spermatozoa (*A* = 5 μm^3^) Based on general velocity, spermatozoa were classified as static (<10 μm/s), slow-medium (10–50 μm/s), or rapid (>100 μm/s). A minimum of 5 fields and 1,000 sperm tracks in each sample chamber were evaluated at 10× magnification on a phase-contrast microscope (image acquisition rate: 25 frames/s). Thawed semen samples were diluted over the range 1:40 to 1:60 (v/v) with PBS (Phosphate-Buffered Saline) and loaded into chamber at 37°C warm plate. The percentage of total motility and the percentage showing progressive motility were recorded. Sperm kinematic parameters, curvilinear velocity (VCL), straight line velocity (VSL), average path velocity (VAP), amplitude of lateral head displacement (ALH), beat crossing frequencies (BCF), and hyperactivity (HPA) were also analyzed. Three progression ratios were calculated from the three velocity measurements as follows: linearity (LIN) of forward progression (LIN = VSL/VCL × 100), straightness (STR = VSL/VAP × 100), and wobble (WOB = VAP/VCL × 100), Mean values of VCL, VSL, VAP, ALH, and BCF parameters indicate the vigor of spermatozoa, whereas LIN, STR, and WOB indicate progressiveness (29).

#### 2.5.2 Sperm quality analysis

##### 2.5.2.1 Sperm viability

Sperm viability was measured by determining the percentage of live sperm using SYBR-14/propidium iodide (PI), as described by Chalah et al. (30) and Santiago-Moreno et al. (31). SYBR-14/PI was prepared by adding 4 μL 0.02 mM SYBR-14 and 2 μL 2.4 mM PI to 100 μL HEPES-buffered medium (containing 130 mM NaCl, 4 mM KCl, 14 mM fructose, 10 mM HEPES, 1 mM CaCl_2_, 0.5 mM MgCl_2_, and 0.1% BSA). The semen samples were prepared for the viability test by diluting 10 μL rooster semen with 200 μL phosphate-buffered saline (PBS). A 10 μL aliquot of the diluted semen was mixed with 20 μL SYBR-14/PI in HEPES-buffered medium and incubated at 20–25°C for 15 min. A sample of 200 spermatozoa were observed under a fluorescence microscope at 400× magnification. Live spermatozoa with intact plasma membranes were stained green by SYBR-14. Live spermatozoa with compromised plasma membranes were stained red and green by SYBR-14 and PI. Additionally, dead spermatozoa with damaged plasma membranes were stained red by PI.

##### 2.5.2.2 Mitochondrial activity

Mitochondrial activity was assessed using JC-1 dye (Molecular Probes, Molecular Probes Inc., Eugene, OR). JC-1 was mixed with SYBR-14 and PI in DMSO at concentrations of 0.153, 0.02, and 2.4 mmol, respectively. This mixture was then combined with HEPES-buffered medium with 1.6 μL JC-1, 1 μL SYBR-14, and 1.6 μL PI. Subsequently, 12.5 μL diluted semen and 25 μL prepared stain mixture were combined and incubated at 20–25°C for 30 min. A fluorescence microscope was used to visualize the mitochondria in 200 spermatozoa tails at 400× magnification. Spermatozoa tails with low mitochondrial function were stained green, while those with high mitochondrial function appeared orange (32).

##### 2.5.2.3 Acrosome integrity

To evaluate the percentage of sperm cells with intact acrosomes, Coomassie blue staining (Merck, Germany) was performed using a modification of the protocol reported by Abouelezz et al. (29). The staining solution (100 mL) was prepared by mixing 22.5 mL 0.5% Coomassie blue, 22.5 mL methanol, 54.75 mL distilled water, and 0.25 mL glacial acetic acid. A drop of 10 μL diluted semen sample was applied on a glass slide, smeared as a circle, and allowed to dry. The smear was subsequently fixed with buffered 4% glutaraldehyde in PBS for 30 min at room temperature (25°C) and air-dried. The slide was stained with Coomassie blue staining solution for 5 min, rinsed with distilled water, and air-dried. Finally, 200 spermatozoa with intact acrosomes were counted under a light microscope with oil immersion at a magnification of 1,000× oil. Spermatozoa exhibiting a hooked, swollen, thinned, or absent acrosome were classified as having no acrosome integrity.

##### 2.5.2.4 Plasma membrane integrity

To evaluate plasma membrane integrity, a 100 mOsm/kg hypoosmotic solution was prepared by dissolving 1 g sodium citrate in 100 mL double-distilled water. A solution containing 3 μL diluted semen and 100 μL hypoosmotic solution was incubated at 20–25°C for 30 min. A drop of the incubated solution was spread on a slide and allowed to dry. Coomassie blue mixed with 0.25% acetic acid staining solution was added to the slide for 2 min. A sample of 200 spermatozoa were observed under a light microscope at 1,000× magnification. Spermatozoa with coiled midpieces and tail segments were classified as having positive plasma membrane integrity, while those without coiled tails were classified as negative (33).

### 2.6 Statistical analysis

Statistical analyses were carried out using SAS (SAS version 9.1cary, NC, United States). The effect of phosphorus and vitamin B12 on sperm motion characteristics and sperm characteristics on time after thawing were analyzed by the general linear mixed model (MIXED). Factors for the statistical model included the experimental groups (control, 0.02, 0.04, 0.06, 0.08, and 0.10% of phosphorus and vitamin B12), time after thawing (0, 15, and 30 min), and interaction between treatment and time after thawing. The following model was applied to analyzed = the data:


$$Y_{ijk}=m+G_{i}+T_{j}+R_{k}+O_{ijk}$$

Where *Y_ijk_* is the response variable, *m* is the overall mean, *G_i_* is the fixed effect of the experimental groups [i.e., 0.00% (control), 0.02, 0.04, 0.06, 0.08, and 0.10% of phosphorus and vitamin B12], *T_j_* is the fixed effect of the time after thawing (i.e., 0, 15, and 30 min), *R_k_* is a random component related rooster and *O_ijk_* is the residual error component. The Thai native rooster was included as a random variable. Least square means were obtained from each class of factor and compared using the least significant test (LSD). *p*-values of <0.05 were considered statistically significant.

## 3 Results

Sperm motility, VCL and HPA were higher in semen supplemented with 0.04% phosphorus and vitamin B12 than in the control (Table 1). VSL, VAP, STR, and BCF were also higher in the 0.08% phosphorus and vitamin B12 supplementation group than in the control group (Table 1). All supplementation doses increased the sperm quality parameters tested compared with the controls (Table 2). In all the experimental groups, sperm motility, kinematic parameters, and sperm quality decreased over time after thawing (Tables 1, 2).

**Table 1:** The effects of phosphorus and vitamin B12 supplementation in semen extender, irrespective of the time elapsed after thawing, as well as the impact of time after thawing, regardless of the concentrations of phosphorus and vitamin B12, on sperm motility and sperm kinematic parameters analyzed via CASA in frozen-thawed rooster semen.

Parameters	Concentrations of phosphorus and vitamin B12,%						SEM*	Min			SEM*
	0.00	0.02	0.04	0.06	0.08	0.10		0	15	30	
Total motility, %	43.2^c^	45.4^b^	47.8^a^	45.8^b^	46.5^ab^	45.8^b^	1.2	49.5^a^	45.6^b^	42.2^c^	1.1
PR**, %	6.9^b^	7.8^a^	8.3^a^	7.9^a^	8.0^a^	8.0^a^	0.4	9.1^a^	7.7^b^	6.8^c^	0.4
VCL, μm/s	48.3^b^	49.4^a^	49.8^a^	49.8^a^	49.5^a^	49.8^a^	0.6	50.8^a^	49.4^b^	48.2^c^	0.6
VSL, μm/s	14.5^b^	14.5^b^	14.6^ab^	14.6^ab^	14.9^a^	14.8^ab^	0.3	15.0^a^	14.6^b^	14.3^c^	0.3
VAP, μm/s	23.7^b^	23.8^b^	24.2^ab^	24.2^ab^	24.4^a^	24.3^a^	0.4	24.8^a^	24.1^b^	23.5^c^	0.3
LIN, %	33.3^ab^	32.7^bc^	32.3^c^	32.9^ac^	33.8^a^	33.3^ab^	0.7	32.6^b^	33.2^ab^	33.4^a^	0.7
STR, %	56.2^b^	56.2^b^	56.2^b^	56.3^b^	57.2^a^	56.6^ab^	0.6	56.3	56.6	56.4	0.6
WOB, %	50.5^ab^	49.7^bc^	50.7^ab^	51.1^ab^	51.9^a^	51.2^a^	0.7	50.0^a^	51.2^b^	51.3^b^	0.6
ALH, μm	3.41	3.36	3.45	3.42	3.49	3.52	0.08	3.46	3.48	3.38	0.06
BCF, beats/s	4.05^b^	4.07^ab^	4.15^ab^	4.10^ab^	4.21^a^	4.06^b^	0.08	4.30^a^	4.08^b^	3.94^c^	0.07
HPA, %	0.90^b^	1.09^a^	1.06^a^	1.08^a^	1.01^ab^	1.08^a^	0.10	1.14^a^	1.08^a^	0.89^b^	0.09

^a, b, c^ Different superscript letters within rows indicate statistically significant difference (*p* < 0.05). *SEM: maximum standard error of the mean and **Progressive motility.

**Table 2:** The effects of phosphorus and vitamin B12 supplementation in semen extender, irrespective of the time elapsed after thawing, as well as the impact of time after thawing, regardless of the concentrations of phosphorus and vitamin B12, on sperm quality in frozen-thawed rooster semen.

Parameters	Concentrations of phosphorus and vitamin B12,%						SEM*	Min			SEM*
	0.00	0.02	0.04	0.06	0.08	0.10		0	15	30	
Viability, %	43.5^e^	46.5^b^	48.8^a^	47.3^bc^	48.2^ac^	45.4^d^	1.0	49.7^a^	46.7^b^	43.5^c^	0.9
Mitochondria, %	37.1^d^	40.6^c^	43.9^a^	42.2^b^	44.4^a^	40.7^c^	1.1	45.0^a^	41.2^b^	38.2^c^	1.1
Acrosome, %	81.7^d^	88.5^b^	91.6^a^	89.0^b^	88.7^b^	84.3^c^	1.2	93.8^a^	87.7^b^	80.4^c^	1.1
Membrane, %	26.2^e^	31.7^c^	35.6^a^	33.6^b^	34.6^ab^	29.6^d^	1.0	35.6^a^	31.8^b^	28.2^c^	1.0

^a, b, c, d, e^ Different superscript letters within rows indicate statistically significant difference (*p* < 0.05). *SEM: maximum standard error of the mean.

### 3.1 Effects of different concentrations of phosphorus and vitamin B12 supplementation and time after thawing on rooster sperm motility

**Figure 1:**
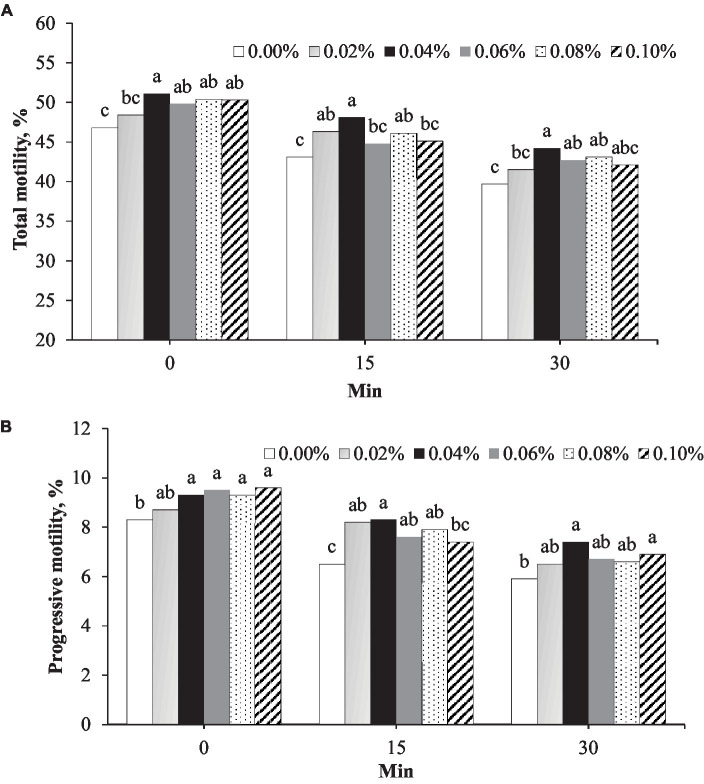
Effect of different concentrations of phosphorus and vitamin B12 supplementation (0.00, 0.02, 0.40, 0.06, 0.08, and 0.10%) on frozen rooster semen at different timepoints after thawing. **(A)** Total motility and **(B)** Progressive motility. ^a, b, c^ Different superscript letters per timepoint indicate statistically significant differences (*p* < 0.05).

Total motility was the highest in semen supplemented with 0.04% phosphorus and vitamin B12 at all timepoints after thawing (Figure 1A). Immediately after thawing (0 min), the total motility in the 0.04% group (51.1%) was greater than that in the control (46.8%, *p* = 0.001) and 0.02% (48.4%, *p* = 0.042) groups (Figure 1A). At 15 min post-thawing, the total motility in the 0.04% group (48.1%) was greater than that in the control group (43.1%, *p* < 0.001). At 30 min post-thawing, the total motility in the 0.04% group (44.2%) was greater than that in the control (39.7%, *p* < 0.001) and 0.02% (41.5%, *p* = 0.042) groups (Figure 1A). At 0, 15, and 30 min after thawing, progressive motility in the group with 0.04% phosphorus and vitamin B12 supplementation was greater than that in the control (*p* < 0.05) (Figure 1B).

### 3.2 Effects of different concentrations of phosphorus and vitamin B12 supplementation and time after thawing on rooster sperm kinematics

At 15 min post-thawing, VCL, VAP, and HPA were greater in the 0.04% phosphorus and vitamin B12 supplementation group than in the control (*p* < 0.05) (Table 3). However, 0.04% phosphorus and vitamin B12 supplementation did not affect sperm kinematics immediately and 30 min after thawing when compared with control group (*p* > 0.05) except VCL at 30 min.

**Table 3:** The interaction between phosphorus and vitamin B12 supplementation to semen extender and time after thawing in frozen-thawed rooster spermatozoa on the sperm trajectory and velocity.

Parameters	0 Min						SEM*	15 Min						SEM*	30 Min						SEM*
	0.00	0.02	0.04	0.06	0.08	0.10		0.00	0.02	0.04	0.06	0.08	0.10		0.00	0.02	0.04	0.06	0.08	0.10	
VCL, μm/s	50.3	50.7	50.6	51.5	50.6	51.3	0.8	47.3^b^	50.3^a^	49.7^a^	49.8^a^	49.8^a^	49.3^a^	0.8	47.3^c^	47.3^bc^	49.1^a^	48.2^abc^	48.1^abc^	48.9^ab^	0.8
VSL, μm/s	15.1^ab^	14.4^b^	15.0^ab^	15.0^ab^	15.4^a^	15.3^a^	0.4	14.2	14.8	14.8	14.4	14.7	14.8	0.4	14.1	14.2	14.0	14.4	14.7	14.3	0.4
VAP, μm/s	24.7^ab^	24.1^b^	24.7^ab^	25.0^a^	25.0^a^	25.1^a^	0.4	23.3^b^	24.5^a^	24.3^a^	24.0^ab^	24.2^a^	24.2^a^	0.4	23.2^ab^	22.9^b^	23.6^ab^	23.6^ab^	23.9^a^	23.7^ab^	0.4
LIN, %	33.2^ab^	31.4^c^	31.7^bc^	32.2^abc^	33.6^a^	33.2^ab^	0.9	33.2	33.2	33.0	32.9	33.4	33.8	0.9	33.5^ab^	33.5^ab^	32.1^b^	33.7^ab^	34.4^a^	33.0^ab^	0.9
STR, %	56.5^ab^	55.3^b^	56.2^ab^	55.7^b^	57.4^a^	56.7^ab^	0.8	56.1	56.5	56.9	56.3	56.7	57.1	0.8	56.1^ab^	56.8^ab^	55.5^b^	56.8^ab^	57.4^a^	56.0^ab^	0.8
WOB, %	48.9^bc^	47.0^c^	50.8^ab^	50.7^ab^	51.6^a^	51.2^ab^	1.0	51.2	51.2	50.9	51.0	51.5	51.5	1.0	51.4	50.8	50.3	51.5	52.5	51.0	1.0
ALH, μm	3.44	3.41	3.47	3.50	3.44	3.51	0.11	3.28^b^	3.42^ab^	3.44^ab^	3.44^ab^	3.69^a^	3.63^a^	0.11	3.52	3.26	3.43	3.32	3.34	3.41	0.11
BCF, beats/s	4.24	4.23	4.34	4.34	4.42	4.24	0.11	4.03	4.13	4.09	4.05	4.08	4.09	0.11	3.89^ab^	3.84^b^	4.01^ab^	3.91^ab^	4.11^a^	3.84^b^	0.11
HPA, %	1.14	1.14	1.14	1.09	1.11	1.20	0.13	0.82^b^	1.20^a^	1.12^a^	1.20^a^	1.02^ab^	1.13^a^	0.13	0.73	0.92	0.91	0.94	0.90	0.90	0.13

^a, b, c^ Different superscript letters within rows indicate significant difference (*p* < 0.05). *Maximum standard error of the mean (SEM).

### 3.3 Effects of different concentrations of phosphorus and vitamin B12 supplementation and time after thawing on rooster sperm quality

**Figure 2:**
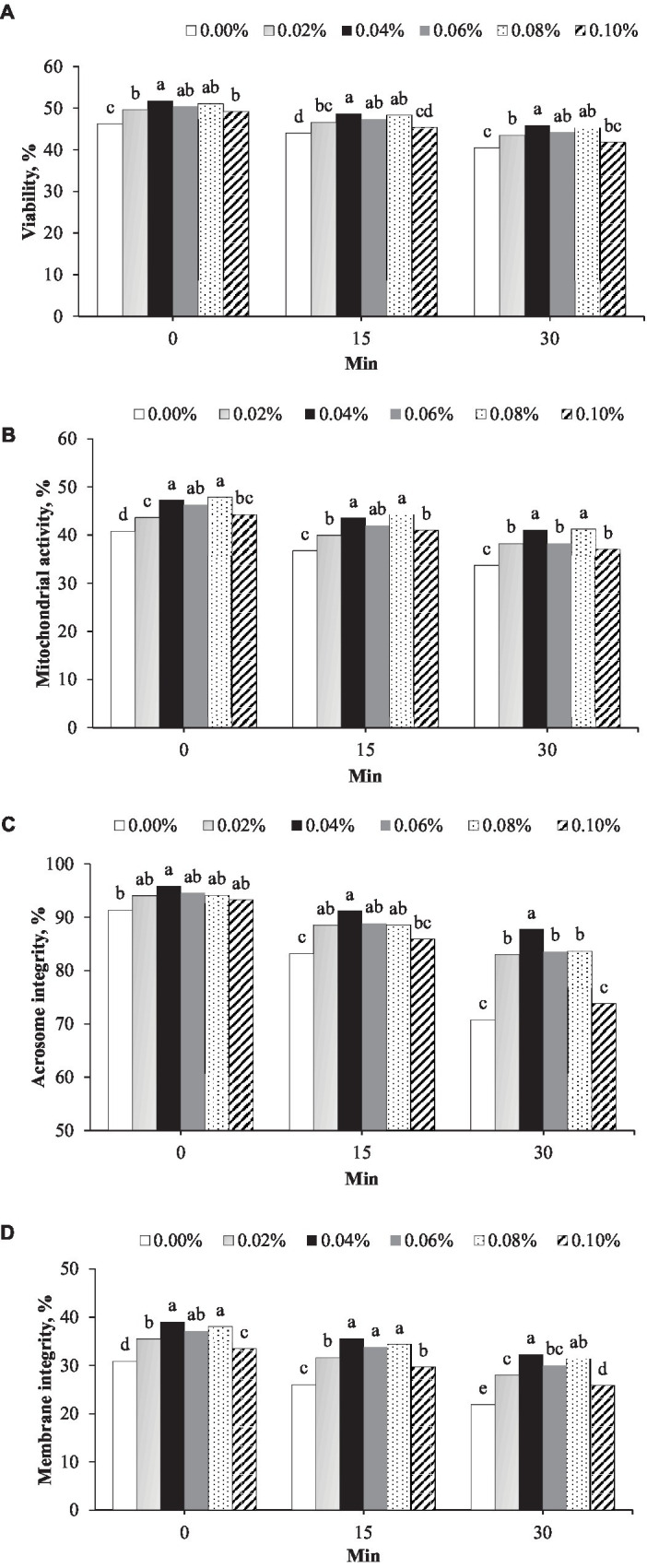
Effect of different concentrations of phosphorus and vitamin B12 supplementation (0.00, 0.02, 0.40, 0.06, 0.08, and 0.10%) on frozen rooster semen at different timepoints after thawing. **(A)** Sperm viability, **(B)** Mitochondrial activity, **(C)** Acrosome integrity, and **(D)** Plasma membrane integrity. ^a, b, c, d, e^ Different superscript letters per timepoint indicate statistically significant differences (*p* < 0.05).

The effects of phosphorus and vitamin B12 supplementation on sperm quality are presented in Figure 2. At all timepoints after thawing, 0.04% supplementation showed the highest sperm viability (Figure 2A). At 0 min post-thawing, the sperm viability in 0.04% (51.8%) was greater than that in the control (46.2%, *p* < 0.001) and 0.02% (49.6%, *p* = 0.026) (Figure 2A) groups. At 15 min post-thawing, sperm viability in the 0.04% group (48.6%) was greater than that in the control (44.0%, *p* < 0.001), 0.02% (46.6%, *p* = 0.038), and 0.10% (45.3%, *p* < 0.001) groups. At 30 min post-thawing, sperm viability in the 0.04% group (45.9%) was greater than that in the control (40.4%, *p* = 0.002) and 0.02% (43.5%, *p* = 0.014) groups (Figure 2A).

At 0 min post-thawing, the mitochondrial activity in the 0.04% phosphorus and vitamin B12 supplementation group (47.2%) was greater than that in the control (40.8%, *p* < 0.001), 0.02% (43.6%, *p* = 0.003), and 0.10% (44.2%, *p* = 0.012) groups (Figure 2B). At 15 min post-thawing, the mitochondrial activity with 0.04% supplementation (43.5%) was greater than that in the control (36.7%, *p* < 0.001), 0.02% (39.9%, *p* = 0.003), and 0.10% (41.0%, *p* = 0.031) groups. At 30 min post-thawing, mitochondrial activity in the 0.04% group (41.1%) was greater than that in the control (33.7%, *p* < 0.001), 0.02% (38.2%, *p* = 0.017), 0.06% (38.2%, *p* = 0.017), and 0.10% (37.0%, *p* < 0.001) groups (Figure 2B).

Acrosome integrity and membrane integrity were the highest in semen supplemented with 0.04% phosphorus and vitamin B12 at all timepoints after thawing (Figures 2C,D). Acrosome integrity was greater in the 0.04% phosphorus and vitamin B12 supplementation group (95.8%) that in the control group (30.9%, *p* = 0.017) immediately after thawing (Figure 2C). At 15 min post-thawing, acrosome integrity in the 0.04% group (88.8%) was greater than that in the control (83.20%, *p* < 0.001) and 0.10% (85.9%, *p* = 0.006) groups. At 30 min post-thawing, acrosome integrity in the 0.04% group (87.7%) was greater than that in the other concentration groups (*p* < 0.05) (Figure 2C). Membrane integrity in the group 0.04% phosphorus and vitamin B12 supplementation (39.0%) was greater than that in the control (30.9%, *p* < 0.001), 0.02% (35.5%, *p* < 0.001), and 0.10% (33.4%, *p* < 0.001) groups at 0 min after thawing, (Figure 2D). At 15 min after thawing, the membrane integrity in the 0.04% group (35.5%) was greater than that in the control (26.0%), 0.02% (31.6%), and 0.10% (29.6%) (*p* < 0.001) groups. At 30 min after thawing, membrane integrity in the 0.04% group (32.3%) was greater than that in the control (21.9%), 0.02% (32.3%), 0.06% (30.0%), and 0.10% (25.6%) groups (*p* < 0.001) (Figure 2D).

## 4 Discussion

To the best of our knowledge, this is the first study to report data on the effects of phosphorus and vitamin B12 supplementation on frozen-thawed rooster semen. Our results demonstrated that supplementation with 0.04% phosphorus and vitamin B12 increased sperm motility, sperm kinematic parameters, and sperm quality. These findings provide a basis for adding phosphorus and vitamin B12 in semen extenders to improve sperm quality in cryopreserved Thai native rooster semen.

Cryopreservation can decrease sperm viability and survival after thawing. This could be attributed to increased lipid peroxidation and sperm acrosome, plasma membrane, DNA, and mitochondria disruption during freezing (12). Previous studies reported that freezing causes the greatest structural damage to the mitochondria, midpiece, and perforatorium (34). Furthermore, rooster spermatozoa exhibit unique characteristics that render them potentially more vulnerable to freezing-induced damage, such as a small cytoplasm, fewer mitochondria, lower cytoplasmic antioxidants, and abundant plasma membrane polyunsaturated fatty acids (9). Additionally, cryopreservation induces the production of reactive oxygen species (ROS) and cellular defense systems, resulting in oxidative stress (35). ROS and free radicals, such as hydrogen peroxide, abolish sperm motility, while hydroxyl radicals can reduce all movement characteristics except straightness and linearity (36).

Thus, some investigators have attempted to improve semen quality by adding antioxidants, inhibitors of lipid peroxidation, and cryoprotectants. Antioxidants, such as resveratrol, lycopene, quercetin, melatonin, vitamin C, E, amino acid, glutathione, and selenium, have been reported to improve frozen rooster semen (37). The amino acid serine decreases lipid peroxidation and improves semen quality and fertilizing ability in frozen-thawed Thai native rooster semen (17). Cryoprotectants act by inducing cell dehydration and reducing intracellular ice crystals formation (38). Sugar is a cryoprotectant that improves semen quality and fertility (18).

Phosphorus enhances sperm motility by acting as substrate for ATP, AMP, and phosphocreatinine production and regulating gluconeogenesis and glycogenesis, which are related to energy metabolism (39, 40). Furthermore, vitamin B12 is a cofactor in the conversion of methylmalonyl coenzyme A (CoA) to succinyl CoA during gluconeogenesis (41). The result of the present study demonstrated that all concentrations of phosphorus and vitamin B12 improved sperm total motility, progressive motility, and sperm kinematics, consistent with the results of a previous study that reported improved motility following phosphorus and vitamin B12 supplementation in chilled boar semen (24). Thus, the supplementation of a combination of phosphorus and vitamin B12 may improve energy efficiency in cryopreserved semen.

The antioxidant activity of vitamin B12 prevents stress-induced membrane lipid peroxidation in sperm, such as in the freezing-thawing process. Many studies also suggest that vitamin B12 plays an important role in spermatogenesis and increases glutathione peroxidase activity (42). Glutathione is a major intracellular antioxidant that protects the cell against oxidative stress. A reduction in spermatozoa glutathione levels after freezing has been reported in bulls, boar, and human semen (43). Our results revealed that 0.04% phosphorus and vitamin B12 supplementation increased sperm viability by 5.3%, mitochondrial activity by 6.8%, sperm plasma membrane integrity by 9.4%, and acrosome integrity by 9.9%. Supplementation with 0.04% phosphorus and vitamin B12 improved VCL and all sperm quality parameters 30 min after thawing. As reported previously, VAP and VCL are good predictors of the ability of spermatozoa to migrate in cervical mucus (44) and are significantly correlated with fertility in bulls (45). Furthermore, the addition of 0.08% phosphorus and vitamin B12 enhanced VAP, LIN, STR, and BCF as well as sperm viability, mitochondrial activity, and membrane integrity 30 min after thawing. These suggest that higher doses of phosphorus and vitamin B12 allow the sperm cell to harness energy to sperm kinematics and quality.

Conversely, the concentration of 0.1% decreased acrosome integrity and membrane integrity, which may be because of potential toxicity at high doses. Thus, optimal concentration of phosphorus and vitamin B12 supplementation could be in the range of 0.4–0.8%.

These findings corroborate those of previous studies, reporting the beneficial effect of vitamin B12 supplementation on semen quality in numerous species (46–48). Moreover, phosphorus and vitamin B12 supplementation obviously improved the quality of chilled semen from Thai Native Chicken (49). Therefore, the addition of an optimized amount of vitamin B12 into the freezing extender could prevent the generation of oxygen radicals, resulting in decreased peroxidation and membrane damage and ultimately improving sperm motility and viability (47). Moreover, the addition of vitamin B12 to bovine semen *in vitro* increased sperm motility, sperm velocity, and proportion of intact sperm by increasing catalase and glutathione reductase activities (50). Therefore, the supplementation of phosphorus and vitamin B12 can improve rooster sperm by protecting the plasma membrane from damage and preventing oxidative stress during semen cryopreservation.

## 5 Conclusion

The supplementation of frozen semen extender with 0.04% phosphorus and vitamin B12 increased sperm motility, kinematics, and quality. Thus, the potential of phosphorus and vitamin B12 supplementation in semen extender in improving frozen rooster sperm quality should be applied in rooster semen cryopreservation.

## Data availability statement

The original contributions presented in the study are included in the article/supplementary material, further inquiries can be directed to the corresponding author.

## Ethics statement

The animal studies were approved by The study protocol was approved by the Institutional Animal Care and Use Committee of the Faculty of Veterinary Science, Chulalongkorn University (Approval number 2131018) and complies with provisions of “The Ethical Principles and Guidelines for the Use of Animals for Scientific Purposes” (edited by the National Research Council of Thailand). The studies were conducted in accordance with the local legislation and institutional requirements. Written informed consent was obtained from the owners for the participation of their animals in this study.

## Author contributions

JS: Conceptualization, Methodology, Software, Writing – original draft, Writing – review & editing. PK: Investigation, Methodology, Writing – original draft. GN: Investigation, Methodology, Writing – original draft. CK: Methodology, Writing – original draft. BO: Investigation, Methodology, Writing – original draft. PS: Investigation, Methodology, Writing – original draft. MN: Conceptualization, Formal analysis, Funding acquisition, Investigation, Methodology, Project administration, Validation, Writing – original draft, Writing – review & editing.
